# Efficacy of oral insulin nanoparticles for the management of hyperglycemia in a rat model of diabetes induced with streptozotocin

**DOI:** 10.25122/jml-2023-0355

**Published:** 2024-02

**Authors:** Ghasak Kais Abd-Alhussain, Mohammed Qasim Yahya Mal-Allah Alatrakji, Shayma'a Jamal Ahmed, Hayder Adnan Fawzi

**Affiliations:** 1 College of Pharmacy, Uruk University, Baghdad, Iraq; 2 College of Medicine, Baghdad University, Baghdad, Iraq; 3 Department of Pharmacy, Al-Mustafa University College, Baghdad, Iraq

**Keywords:** nanoparticles, animal model, chitosan, insulin, hyperglycemia, DM, diabetes mellitus, NaCMC, sodium carboxymethyl cellulose, NPs, nanoparticles, PDI, polydispersity index, PLGA, poly(lactic-co-glycolic acid), STZ, streptozotocin, TMC, N-trimethyl chitosan chloride, TPGS, D-α-Tocopherol polyethylene glycol succinate

## Abstract

Insulin is the cornerstone of treatment in type 1 diabetes mellitus. However, because of its protein structure, insulin has to be administered via injection, and many attempts have been made to create oral formulations, especially using nanoparticles (NPs). The aim of this study was to compare the hypoglycemic effect of insulin-loaded NPs to that of subcutaneous insulin in an in vivo rat model of diabetes. We used biodegradable D-α-tocopherol polyethylene glycol succinate-emulsified, chitosan-capped poly(lactic-co-glycolic acid) NPs loaded with soluble human insulin in a dose of 20 IU/kg body weight, and examined the physical characteristics of NPs in vivo and in vitro. Serum glucose levels were reduced after 6 h, but the difference was not significant compared to subcutaneous insulin; at 12 h and 24 h, insulin levels were significantly higher in rats treated with NPs than in rats treated with subcutaneous insulin. There was no significant difference in serum insulin levels at 12 h and 24 h compared to non-diabetic rats. Our findings suggest that chitosan-based NPs are able to maintain good glycemic control for up to 24 h and can be considered a potential carrier for oral insulin delivery.

## INTRODUCTION

Diabetes mellitus (DM) is a significant health issue with a rising global prevalence [1,2]. Approximately 1.4 million Iraqis suffer from DM [3], the prevalence of type 2 DM in Iraq ranging from 9% to 14% [3]. A study conducted on over 5,400 individuals aged 19–94 years from the city of Basrah, located in Southern Iraq, revealed an age-adjusted prevalence of DM of 19.7% [4]. Owing to the increased burden of DM, there is growing interest to examine the impact of DM and its association with other conditions in Iraqi patients [5–10].

Current therapies of insulin-dependent type 1 DM (T1DM) are directed towards the substitution of insulin, either orally [11–14] or non-orally [15–17]. However, insulin delivered through a controlled-release system has significant shortcomings because of aggregation and precipitation. Owing to the short plasma half-life of insulin of 3–10 min and the fact that basal insulin returns to normal after 2–4 h following a meal [18,19], insulin exhibits poor physicochemical characteristics. The main obstacles to unmodified intake and transport of insulin into the circulatory system are low pH and protease degradation, further exacerbated by its high molecular mass and hydrophilic character [20–22].

Patients with DM favor the oral administration of insulin because it is easier and more convenient. Several insulin transporters, such as polymeric nanoparticles (NPs), liposomes, and lipids, have been successfully synthesized in recent years [23–25]. Biodegradable polymer microspheres or NPs have shown promise in the oral administration of protein and peptide medicines. Compared to microspheres, the advantage of NPs is that they enter the gastrointestinal tract and are taken up by the M cells of Peyer’s patches, the main entry point for NP absorption [26]. Poly(lactic-co-glycolic acid) (PLGA) NPs have gained significant attention owing to their ability to encapsulate and deliver drugs in a controlled manner effectively. As a result, PLGA NPs have been extensively used as nanocarriers [27]. However, they have important shortcomings, such as selective interaction with mucosal surfaces [28,29].

N-trimethyl chitosan chloride (TMC) is a partially quaternized chemical derived from chitosan through reductive methylation [30]. In environments with a neutral pH, where chitosan is not soluble and does not improve permeability effectively, TMC retains its positive charge, high solubility, and ability to promote permeation [31,32]. Insulin-loaded TMC NPs have a positive charge and a mucoadhesive nature, and are able to penetrate the intestinal epithelium [33]. In environments with high ionic strength, such as the intestinal fluid, the electrostatic connection between the negatively charged insulin and positively charged TMC weakens, leading to rapid insulin release from TMC NPs. Approximately 50% of the insulin contained within TMC NPs is released in PBS within 1 h [34].

Although TMC NPs produced using the ionic crosslinking method are able to penetrate the intestinal epithelium, this ability can be further improved [33]. The aim of this study was to compare the efficacy of insulin-loaded, D-α-tocopherol polyethylene glycol succinate (TPGS)-emulsified, chitosan-capped PLGA NPs to that of subcutaneous insulin in an in vivo rat model of diabetes and assess the physical characteristics of synthesized NPs.

## MATERIAL AND METHODS

### Study design and settings

The study included 32 male adult albino Wistar rats weighing 180–250 g, marked on various body parts for easy recognition. Mean body weight variation did not exceed 20%. The rats were obtained from the Animal Facility of the Biotechnology Research Center of Al-Nahrain University, Baghdad, Iraq. Before the investigation, the rats were kept in a temperature-controlled setting (22 ± 2 °C) with a reversed diurnal period (12/12 h) and habituated for 7 days. Throughout the research, the animals were maintained on a standard pellet diet and access to water ad libitum supplied by the Biotechnology Research Center of Al-Nahrain University.

Diabetes was induced with streptozotocin (STZ) (Sigma Chemicals). Before the experiment, the serum glucose level of the rats was measured to exclude the possibility of spontaneous diabetes. Only rats with glucose levels of 50–70 mg/dl were included, as described in the literature [35]. The induction process lasted 72 h, the experiment being conducted on day 4.

**Figure 1:**
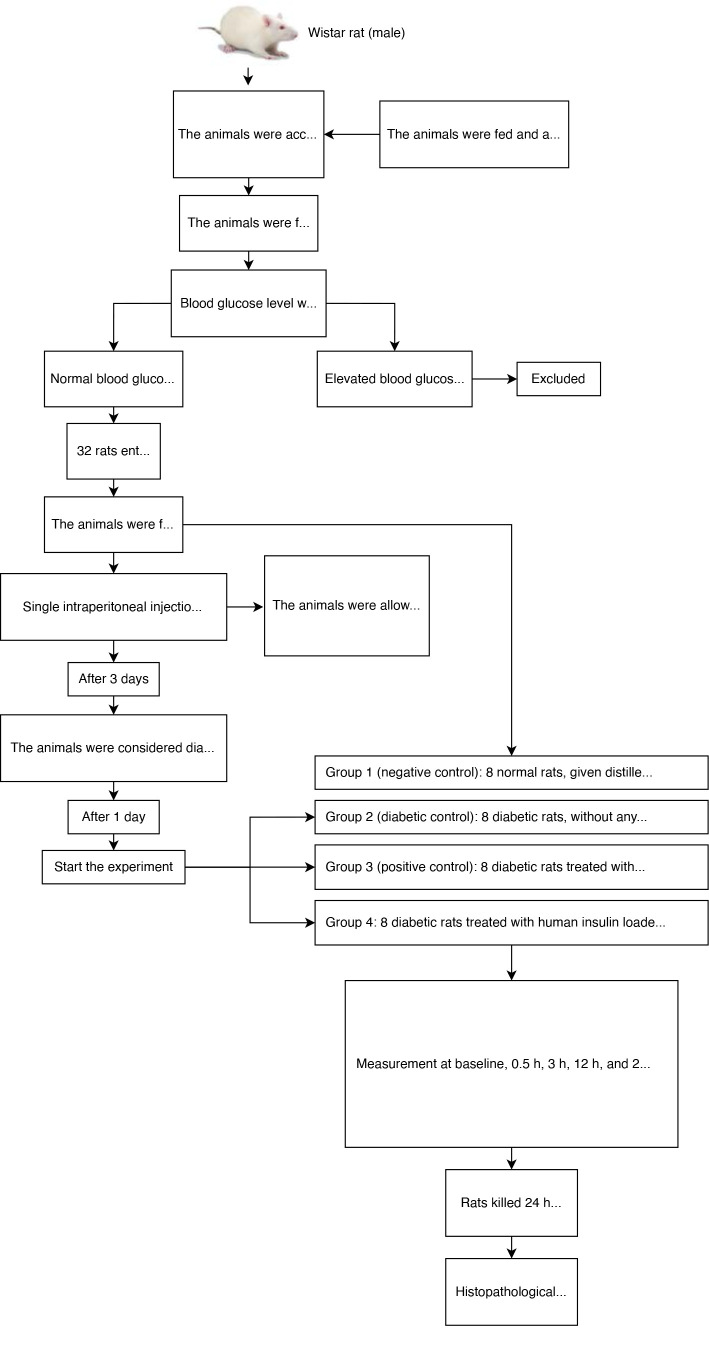
Flow chart of the study

The rats were allocated randomly into four equal groups, as follows: group 1 acted as the negative control (*n* = 8); these animals did not undergo induction and were supplied with distilled water orally. Group 2 acted as diabetic control (*n* = 8); these rats were induced T1DM by intraperitoneal STZ injection and left without DM treatment during the experimental period. Rats in group 3 (*n* = 8) were induced T1DM by intraperitoneal STZ injection and were administered human insulin subcutaneously at a dose of 1 IU/kg body weight, as described in the literature [36]. Rats in group 4 (*n* = 8) were induced T1DM by intraperitoneal STZ injection and treated with human insulin coated with PLGA-chitosan-TPGS NPs at a dose of 20 IU/kg body weight. All drugs (except for subcutaneous insulin in group 3) were given orally. In the case of group 4, the polymer was suspended in a sodium carboxymethyl cellulose (NaCMC) solution containing 1% (w/v) sodium carboxymethyl cellulose (Figure 1).

### Induction of diabetes in rats

The rats were acclimated and subjected to an overnight fast. Subsequently, they were administered a single intraperitoneal injection of a freshly prepared solution of STZ (60 mg/kg body weight) [37] in a 0.1 M cold citrate buffer (pH 4.5). During the induction, the rats were provided with a 5% glucose solution over night to mitigate severe hypoglycemia resulting from the excessive release of insulin generated by the administration of STZ. The rats were classified as diabetic if their blood glucose levels exceeded 250 mg/dl at 72 h after STZ administration. The administration of experimental therapy started on day 4 after the STZ injection [38]. Rats with no diabetes induction were subjected to the same protocol, but instead of STZ, they were administered intraperitoneal injections of 0.9% saline solution. At the end of this phase, animals that were not subjected to induction and had blood glucose levels of <100 mg/dl were included in the negative control group.

### Preparation of polymeric NPs

#### Human insulin, PLGA, chitosan, and TPGS NPs

**Figure 2:**
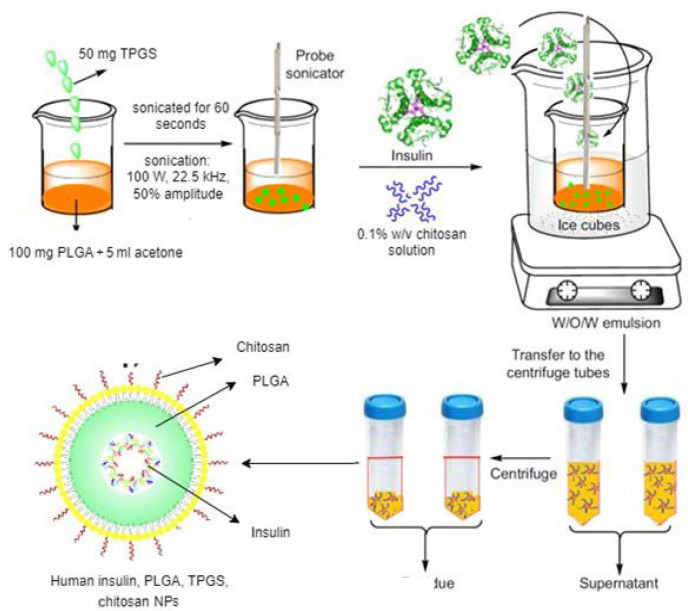
Synthesis of insulin-loaded NPs

A solution was prepared by dissolving 100 mg of PLGA copolymer (PLGA 50/50) and 50 mg of TPGS in 5 ml of acetone. The solution underwent sonication for 60 s using a Sonicator S-4000 probe sonicator (Misonix) (100 W, 22.5 kHz, 50% amplitude) while consistently stirring at 4 °C. A volume of 2 ml of human insulin (100 IU/ml, Novo Nordisk) was added to the solution with continuous stirring, and the solution was subjected to sonication for another 60 s. The solution was kept in an ice bath, forming a water-in-oil (W/O) emulsion, then it underwent sonication again for 180 s, using an equivalent volume of a 0.1% w/v chitosan solution prepared by dissolving chitosan in 1% v/v acetic acid. The sonication process was carried out under continuous stirring at a speed of 500 r.p.m. for 1 h. Following the completion of the coating reaction, the mixture underwent a purification process involving three cycles of centrifugation; this was done to separate the NP pellet from the supernatant, which contained the leftover chitosan solution (Figure 2).

### Nanoparticle characterization

Particle size and distribution, including mean diameter and polydispersity index (PDI), were assessed using dynamic light scattering with a NanoBrook 90Plus particle analyzer (Brookhaven Instruments) at the Nanotechnology and Advanced Materials Research Centre of the University of Technology, Baghdad, Iraq. All measurements were done in triplicate.

Zeta potential (ζ) was assessed using a NanoBrook ZetaPlus analyzer (Brookhaven Instruments) using electrophoretic light scattering (ELS), as described previously [39], at the Nanotechnology and Advanced Materials Research Centre of the University of Technology, Baghdad, Iraq.

Encapsulation efficiency was evaluated using the indirect technique [40] (Equation 1). In brief, the NPs were centrifuged at 20,000 r.p.m. for 10 min, and the insulin content in the supernatant was determined using an established high-performance liquid chromatography (HPLC) technique. Insulin retention time was 7.9 ± 0.05 minutes with an injection volume of 30 µl. Insulin had a maximum wavelength of detection (λ_max_) of 210 nm. In total, 5 mg of NPs were dissolved in 5 ml of acetone using vortex for 15 min before centrifugation at 12,000 r.p.m. at 4 °C for 10 min. At 210 nm, the insulin concentration in the supernatant was measured using a PerkinElmer λ35 spectrophotometer (PerkinElmer), as described in the literature [38].


$$1)\;EE=\frac{Initial\;amount\;of\text{ insulin - }amount\;of\;insulin\;unentrapped}{Intial\;amount\;of\;insulin}x100$$

### In vitro drug release from NPs

We recorded the in vitro release profile of NPs in PBS at 37 °C. The amount of released insulin was monitored spectrophotometrically at 210 nm, as described in the literature [38]. Insulin release was maintained for 24 h.

### HPLC

As described previously by Zhang *et al*. [41], the NPs were isolated from the aqueous suspension medium using ultracentrifugation at 40,000g and 10 °C for 30 min. The quantity of free insulin in the clear supernatant remaining after centrifugation was measured using a HP1100 HPLC (Angilent). The experimental setup involved an HPLC system consisting of a pump and a UV–V detector. Insulin elution was performed using a Hypersil ODS C18 column with dimensions of 200 × 4.6 mm, at 5 µm. The mobile phase consisted of a combination of water, acetonitrile, and trifluoroacetic acid in a ratio of 82:18:0.3. The flow rate used in the experiment was 1.0 ml/min.

Detection was performed at a wavelength of 210 nm. A volume of 20 µl of the transparent supernatant was introduced into the HPLC apparatus. The peak area of insulin was recorded, and the insulin concentration was calculated from a standard curve.

### Sample size and randomization

Sample size was computed using G Power [42] based on Cohen’s principles [43]. A table of random integers was used to construct the groupings at random. The animals were placed in labeled containers and given tags for easy recognition [44].

### Outcome measures

On the day of the experiment, blood samples were drawn at regular intervals (at baseline and after 30 min, 3 h, 12 h, and 24 h) for the biological study of serum glucose (ACCU-CHEK Performa) and insulin levels (ELISA). After the experiment, total cholesterol, triglyceride, urea, creatinine, aspartate aminotransferase (AST), and alanine aminotransferase (ALT) levels were measured. Following completion of therapy, all animals were fasted for 12 h and anesthetized intraperitoneally with 80 mg/kg of ketamine and 10 mg/kg of xylazine. Following total anesthesia, all rats were killed by exsanguination (cardiac puncture), a procedure suitable for tissue harvest and conservation [45,46].

### Hormonal and biochemical analysis

Biochemical parameters were analyzed using specific kits by Biolabo: cholesterol with CHOLESTEROL CHOD PAP, triglycerides with TRIGLYCERIDES GPO Method, ALT with ALT-TGP Colorimetric Method, AST with AST-TGP Colorimetric Method, urea with UREA Colorimetric Method, and creatinine with CREATININE Colorimetric Method. Insulin levels were measured using the sandwich-ELISA method (Rat Insulin, INS ELISA Kit, Sunlong).

### Scanning electron microscopy

Surface morphology was examined at the Nanotechnology and Advanced Materials Research Center at the University of Technology, Baghdad, Iraq, using a TESCAN MIRA3 scanning electron microscope (Tescan), at high resolution with a scale bar of 200–500 nm [38].

### Statistical analysis

Statistical analysis was carried out using GraphPad Prism v.10.0.0 for Windows (GraphPad Software). Continuous variables were assessed using analysis of variance (ANOVA) (normal distribution was assessed using the Anderson–Darling test) and pairwise comparisons were made using the post-hoc Tukey test. A two-tailed *P* value of <0.05 was considered statistically significant.

## RESULTS

### Characterization of NPs

**Figure 3:**
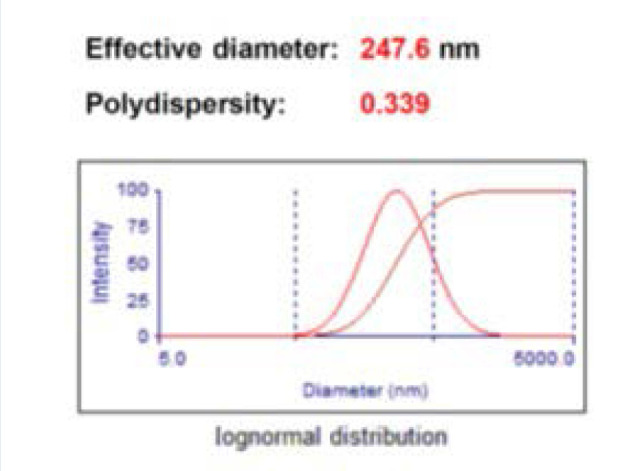
Assessment of the physical characteristics of NPs

**Figure 4:**
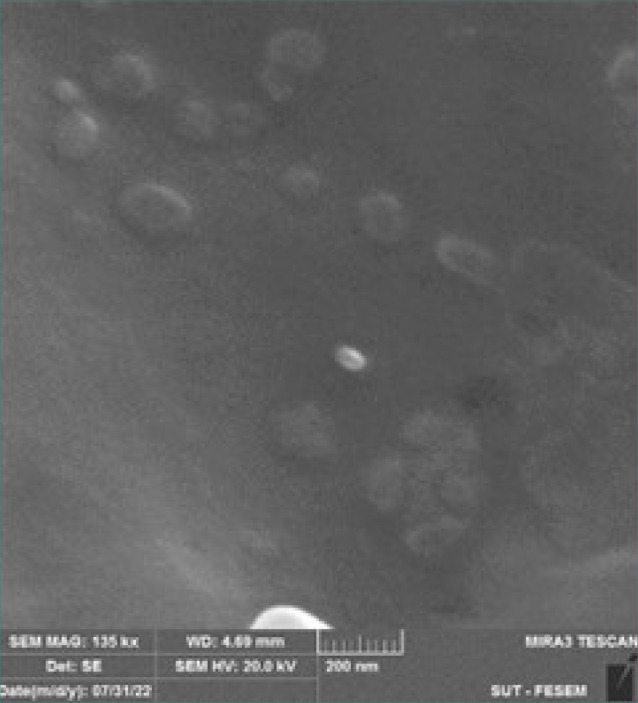
SEM images of the insulin-loaded NPs

Particle size, zeta potential, entrapment efficiency, and PDI are presented in Figures 3 and 4, and Table 1.

**Table 1:** Assessment of NP characteristics

Name of polymer	Particle size (nm)	Zeta potential (mV)	EE (%)	PDI
NP	247.6 ± 7.3	14.4 ± 2.1	47.3 ± 1.3	0.339 ± 0.02

### In vitro release

**Figure 5:**
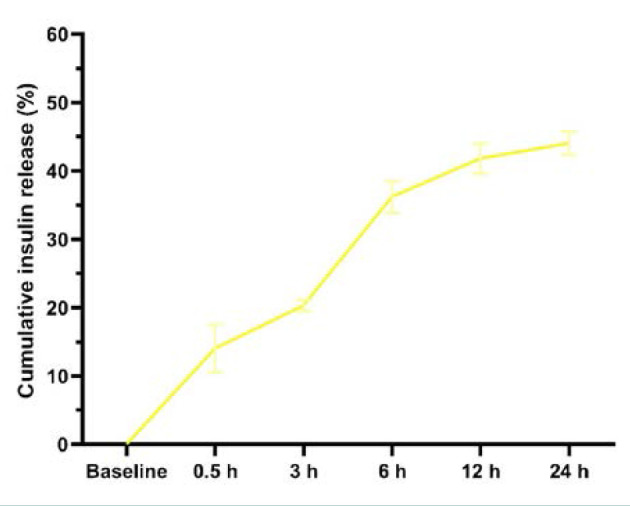
Cumulative insulin release profile for insulin-loaded NPs (pH = 7.4)

The cumulative insulin release was almost linear throughout the assessment (Figure 5).

### In vivo study

#### Effect on blood glucose levels

**Figure 6:**
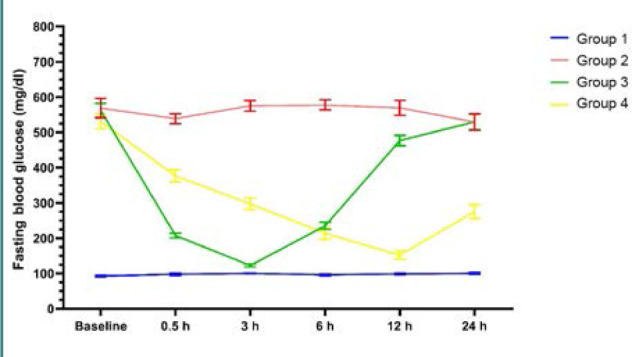
Assessment of fasting blood glucose throughout the study period

**Figure 7:**
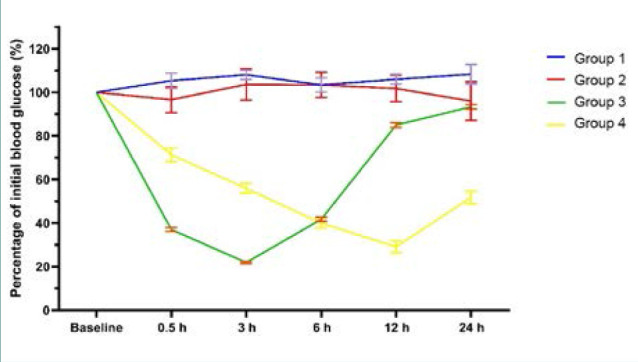
Percentage of initial blood glucose (%)

There were no significant differences between the serum glucose levels of rats treated with subcutaneous insulin and NPs after 6 h. However, glucose levels remained significantly lower in rats treated with NPs than in those treated with subcutaneous insulin at 24 h. The largest effect of NPs on glucose levels could be observed after 12 h and was not significantly different than in the control group (Figures 6 and 7, and Table 2).

**Table 2:** Assessment of fasting blood glucose

	Baseline	0.5 h	3 h	6 h	12 h	24 h
Group 1	93.11 ± 5.9^a^	97.89 ± 8.53^a^	100.34 ± 3.11^a^	96.01 ± 7.16^a^	98.54 ± 6.19^a^	100.33 ± 8.17^a^
Group 2	568.4 ± 77.9^b^	538.91 ± 40.24^d^	575.43 ± 42.77^c^	577.80 ± 40.41^c^	569.43 ± 60.99^d^	529.11 ± 66.5^c^
Group 3	562.4 ± 57.2^b^	207.53 ± 19.91^b^	122.85 ± 13.07^a^	234.85 ± 29.09^b^	477.10 ± 42.02^c^	525.86 ± 63.18^c^
Group 4	534.6 ± 72.2^b^	379.27 ± 55.23^c^	308.87 ± 49.33^b^	215.42 ± 50.02^b^	148.83 ± 29.71^a^	270.20 ± 63.26^b^
*P* value	<0.001	<0.001	<0.001	<0.001	<0.001	<0.001

In each column, there are statistically significant differences between values marked with different letters, and no statistically significant differences between values marked with identical letters (*P* > 0.05). One-way ANOVA, post-hoc Tukey test

#### Effect on insulin level

After 6 h, serum insulin levels were significantly higher in rats treated with NPs than those treated with subcutaneous insulin, and this effect was maintained after 24 h (Table 3).

**Table 3:** Assessment of fasting insulin

	Baseline	0.5 h	3 h	6 h	12 h	24 h
Group 1	3.19 ± 0.12^a^	3.26 ± 0.18^d^	3.11 ± 0.11^c^	3.31 ± 0.118^c^	3.19 ± 0.20^c^	3.20 ± 0.19^c^
Group 2	0.51 ± 0.21^b^	0.41 ± 0.13^a^	0.35 ± 0.11^a^	0.45 ± 0.12^a^	0.43 ± 0.14^a^	0.36 ± 0.10^a^
Group 3	0.36 ± 0.17^b^	1.76 ± 0.18^c^	3.12 ± 0.29^c^	0.52 ± 0.15^a^	0.47 ± 0.19^a^	0.58 ± 0.11^a^
Group 4	0.58 ± 0.12^b^	1.00 ± 0.25^b^	2.01 ± 0.31^b^	2.12 ± 0.48^b^	2.53 ± 0.36^b^	1.70 ± 0.27^b^
*P* value	<0.001	<0.001	<0.001	<0.001	<0.001	<0.001

In each column, there are statistically significant differences between values marked with different letters, and no statistically significant differences between values marked with identical letters (*P* > 0.05)

#### Assessment of biochemical parameters

Changes in biochemical parameters after the end of the experiment are presented in Table 4.

**Table 4:** Assessment of biochemical parameters

Groups	Triglyceride	Total cholesterol	ALT	AST	Urea	Creatinine
Group 1	131.65 ± 16.69^a^	68.20 ± 1.83^a^	49.59 ± 8.04^a^	29.18 ± 5.60^a^	33.33 ± 5.22^a^	0.51 ± 0.08^a^
Group 2	530.90 ± 19.96^d^	119.53 ± 2.32^d^	124.28 ± 21.70^b^	86.48 ± 18.99^b^	100.39 ± 16.76^d^	2.23 ± 0.43^d^
Group 3	481.34 ± 15.60^c^	109.50 ± 2.90^c^	106.41 ± 19.24^b^	67.00 ± 18.99^b^	77.34 ± 16.83^c^	1.78 ± 0.28^c^
Group 4	329.20 ± 14.97^b^	79.87 ± 2.86^b^	64.50 ± 12.68^a^	47.88 ± 9.89^a^	55.78 ± 15.04^b^	1.09 ± 0.24^b^
*P* value	<0.001	<0.001	<0.001	<0.001	<0.001	<0.001

In each column, there are statistically significant differences between values marked with different letters, and no statistically significant differences between values marked with identical letters (*P* > 0.05)

## DISCUSSION

### Efficacy of NPs

PLGA copolymers have been frequently used as an insulin delivery system for oral administration [47]. Particle size has a significant impact on the intestinal absorption of polymeric NPs, the absorption of NPs with a diameter of <500 nm being hundreds of times higher than that of particles with a diameter of >500 nm [48]. In this study, the particle size of NPs was 247.6 ± 7.3 nm, which resulted in enhanced systemic absorption, as shown in previous studies [36,41,48].

The rate of insulin secretion exhibited a fast initial increase within the first 6 h, followed by a subsequent deceleration throughout the remaining duration of the study. This finding suggests that insulin was present on the surface of the NPs. During the production process of chitosan PLGA NPs, insulin can be included in the coating process; this involves enveloping negatively charged PLGA NPs with positively charged chitosan. The observed decrease in insulin discharge suggests that a significant portion of the insulin is enclosed within the matrix of the NPs [41].

The zeta potential of the CS-PLGA-NPs exhibited a positive charge (14.4 ± 2.1 mV), which can be attributed to the presence of chitosan covering. NPs with a positive charge have a higher degree of efficacy in interacting with biomembranes than those with a negative charge. The smooth muscle cells in the gastrointestinal tract contribute to its overall surface charge of approximately −50 mV. Cell permeability and transport efficiency are altered by interactions that involve positively charged NPs and negatively charged cells, which occur as a result of electrostatic forces [49].

TPGS is an effective emulsifying agent that increases solubility and bioavailability. The analytical settings, including sonication duration, sonication rate, centrifugal time, and centrifugal speed, have been tuned so that the series yields NPs of almost the same size. The particle diameter should be <500 nm to maximize interaction with the intestinal mucosa and enhance insulin absorption via the digestive system [50].

In this study, insulin-loaded NPs were administered to rats with induced diabetes. The NPs exhibited efficacy in decreasing blood glucose levels, with the hypoglycemic effect persisting for 24 h and manifesting as early as 6 h. Other studies have reported similar findings [41,51,52].

Delivered orally, insulin degrades in the stomach due to the presence of gastric proteolytic enzymes [53]. Therefore, oral insulin should be enclosed in a matrix-like structure for protection against digestive enzymes; this is made possible by encapsulating the insulin molecules in a chitosan NP matrix. Owing to the mucoadhesive and soluble qualities of chitosan and its ability to prevent protein aggregation, this formulation remains concentrated for a longer period in the small intestine, resulting in delayed absorption and extended availability in the circulation [54].

In our study, the duration of the glucose-lowering effect achieved through the oral administration of insulin-loaded NPs was much longer than the effect of free insulin. A comparative analysis of the pharmacological effects of insulin-loaded NPs and orally administered free insulin revealed that NPs exhibited enhanced insulin absorption, potentially attributed to their ability to extend the retention time of insulin at the intestinal mucosa and facilitate its internalization by enterocytes [55].

Investigations regarding the effect of oral insulin using PLGA–hypromellose phthalate 55 (Hp55) NPs [56], chitosan-coated PLGA NPs [57], and sodium deoxycholate complex-loaded PLGA NPs revealed that each of these complexes is effective in lowering blood glucose levels and their effect is similar to the that of the NPs analyzed in this study with human insulin at a dose of 20 IU/kg body weight [58].

Wu *et al*. used Hp55-coated capsules with insulin-loaded PLGA/RS (Eudragit RS, Evonik Industries) NPs to investigate their efficacy in reducing blood glucose levels in a rat model of diabetes induced with STZ and reported a sustained glucose-lowering effect [59]. Similarly, Kumar *et al*. found that insulin-loaded PLGA NPs effectively decreased serum glucose levels in rats with diabetes induced with STZ [60].

### Assessment of safety

We observed the most substantial decrease in triglyceride and total cholesterol levels in the group of rats treated with NPs. Consistent with the results of previous studies [38,61], triglyceride and total cholesterol levels were significantly higher in diabetic rats than in non-diabetic rats. There were no significant differences in ALT and AST levels between rats treated with NPs and non-diabetic rats. Urea and creatinine levels were higher in rats treated with NPs compared to non-diabetic rats, whereas ALT, AST, urea, and creatinine levels were lower in rats treated with NPs compared to diabetic rats treated with subcutaneous insulin and rats without treatment.

The increase in blood urea concentration observed in diabetic rats may be attributed to a decrease in the level of plasma proteins, an increase in the level of circulating amino acids, and hepatic deamination. Renal impairment could also be a contributing factor [62]. Creatinine is synthesized through the nonenzymatic degradation of creatine phosphate within myocytes. Fluctuations in creatinine levels are thought to be an indicator of compromised glomerular function, and these fluctuations are linked to the nephrotoxic effects of diabetes [63]. NPs reduced all of these effects.

ALT and AST are mostly endogenous enzymes within cells. However, they can be released into the bloodstream due to cellular damage, making them valuable diagnostic biomarkers, particularly for liver injury. In diabetes, aminotransferase activity is increased [38,64]. As shown in the current study, insulin-loaded NPs may protect against STZ-induced diabetic liver damage.

## CONCLUSION

In this study, chitosan-based PLGA NPs produced a glucose-lowering effect that was maintained for 24 h. In terms of safety, NPs reduced total cholesterol, triglyceride, ALT, AST, urea, and creatinine levels compared to those in non-treated diabetic rats. These findings suggest that chitosan PLGA –TPGS NPs are an effective and safe candidate for oral insulin delivery.

## Data Availability

Data analyzed in this article are available in the Zenodo repository at https://zenodo.org/records/8218984, with the DOI 10.5281/zenodo.8218984. The dataset contains the data of the animal studies and physicochemical outcomes, the ethical approval of the study, figures, and the ARRIVE checklist for reporting on animal studies.
